# Photoperiod-Induced Transcriptional Response in the Suprachiasmatic Nucleus

**DOI:** 10.1177/07487304251328461

**Published:** 2025-03-24

**Authors:** Nemanja Milićević

**Affiliations:** Faculty of Medicine and Health Technology, Tampere University, Tampere, Finland

The ability of the suprachiasmatic nucleus (SCN) to encode photoperiod information plays a major role in regulating seasonal rhythms. Day-length information is integrated via the plasticity of the SCN neuronal network and further transmitted to other brain nuclei (Coomans et al., 2015), and ultimately to the pineal gland, which secretes melatonin at night, with a duration proportional to night length. However, little is known about the transcriptional changes that underly the SCN network remodeling in response to photoperiod changes. Early studies reported that the daily pattern of clock gene expression varies with photoperiod in the hamster SCN (Tournier et al., 2003). Recently, Cox and colleagues (Cox et al., 2024) further tackled this question and provided evidence for photoperiod-induced transcriptional plasticity of the SCN. The experiments were conducted using melatonin proficient mice (C3Hf^+/+^), a strain known to exhibit plasticity in the SCN circuitry in response to changes in day-length (Giannoni-Guzmán et al., 2021). The authors used RNA sequencing to study the effects of photoperiod length on the SCN transcriptome of mice. They found that 15% of transcripts were rhythmically expressed in both photoperiods. Substantially more rhythmically expressed genes were found in the SCNs of mice obtained under a short photoperiod (32.3% of all detected genes) compared to the ones kept in a long photoperiod (19.2% of all detected). However, Cox and colleagues report that the timing of expression peaks for most genes was advanced in long compared to short photoperiods (Cox et al., 2024).

To probe the transcriptome differences, Cox and colleagues performed differential expression (DE) analysis of SCNs obtained in long versus short photoperiods. They found 972 (~5.8% total) significantly downregulated and 546 (~3.2% total) significantly upregulated genes in long photoperiod compared to the short one. The differentially expressed genes (DEGs) and the direction of their expression changes are consistent with prior studies. Among the affected pathways were the ones related to retinoic acid signaling (i.e. *Lrat*) and the photoperiod endocrine system (*Tshβ, Npvf, Dct, Dio, Tafa3* and *Slc16a2*). Gene-set enrichment analysis revealed that the DEGs were enriched in terms related to locomotor rhythms, SCN light signaling pathways, peptide hormone response, as well as GABA, chloride, potassium, and calcium transport. Notably, 33 enriched gene-sets related to locomotor activity in DEGs between long and short photoperiods. It is plausible that these pathways might be involved in seasonal adaptations underlying a higher metabolic rate and locomotion in long photoperiod, and an energy-conserving state with shorter activity bouts in short photoperiod.

The authors reported that the rhythms of clock gene expression were advanced for 2 to 5 h in long photoperiod, with numerous clock genes losing rhythmic power in this condition. This photoperiod induced an increase in the expression of the circadian-associated gene *Timeless*, a gene whose function remains unknown in mammals. However, the authors did not find any significant differential gene expression in clock genes between long and short photoperiods. The phase advancement of SCN rhythms in long-day conditions is consistent with the group’s prior work (Ciarleglio et al., 2011), but contrast with earlier work showing that many clock genes have a delayed phase in long compared to short photoperiod in the SCN of Syrian hamsters (Tournier et al., 2003).

Cox and colleagues also investigated the expression profiles of neuropeptide signaling genes. They found several DEGs between the photoperiods, among which *Prokr2* and *Cck* had the highest fold change. The authors compiled a light-response gene-set based on published data and found significant DE of 166 of these 959 light-response genes across photoperiods. Among these genes, *Dusp4*, *Rasd1*, and *Gem* were highlighted due to their known functions in the light transduction pathway and light-mediated phase response in the SCN. Conversely, substantially less DE was observed in genes and pathways which are known to be affected by changes in daylength. Based on these findings, the authors proposed a potential mechanism underlying transcriptional plasticity to photoperiod length involving DE of *Dusp4*, *Rad1*, and *Gem* in long photoperiod, leading to decreased MAPK/ERK activation, CREB/CRE-driven *Period* gene induction, and the SCN phase-shifting light response (Cox et al., 2024).

As described in the methods, the mice in this study were bred as a second generation in long or short photoperiod. They were kept in their photoperiods from birth until P50. The authors argued that this approach minimizes *in utero* and maternally transmitted photoperiod signaling (Ciarleglio et al., 2011). It is plausible that these responses may not be reversible in such long-term and cross-generational placement in long or short photoperiods. Thus, it would be interesting to probe the transcriptome of the SCN to photoperiod inversion. Furthermore, more DEGs were expected in GABA and ion channel-related terms between photoperiods (Cox et al., 2024). It was argued that these changes are likely SCN region-specific, and thus, not detectable in whole SCNs due to the limitations of the bulk-sequencing methodology. This could be addressed by using spatial and/or single-cell transcriptomics. Those approaches have the sensitivity to detect DEG in small populations of SCN cells and to reveal the identities of those SCN cell types.

The group extensively utilized the melatonin proficient mouse line (C3Hf^+/+^) for studying the molecular and neural network plasticity of the SCN in mediating photoperiod adaptations (Giannoni-Guzmán et al., 2021; Cox et al., 2024). However, others described SCN network and molecular plasticity in response to changes in daylength in melatonin deficient C57BL/6 mice (Rohr et al., 2019; Joye et al., 2023). Thus, it is plausible that there is similar photoperiod-induced transcriptional plasticity in the SCN in melatonin deficient mice.

Overall, this work presents fascinating insights into the transcriptional hallmarks of photoperiodicity in the murine SCN. The detailed supplementary material and online resource (http://circadianphotoperiodseq.com) provide ample opportunity for future inquiry. The analysis was performed using a myriad of bioinformatics tools and existing SCN transcriptomics databases that strengthen the conclusions of this paper. The extensive datasets will facilitate future mechanistic studies in characterizing genes critical for SCN photoperiod plasticity.
